# Multimorbidity and depressive symptoms in older adults and the role of social support: Evidence using Canadian Longitudinal Study on Aging (CLSA) data

**DOI:** 10.1371/journal.pone.0276279

**Published:** 2022-11-10

**Authors:** Lixia Zhang, Shahin Shooshtari, Philip St. John, Verena H. Menec

**Affiliations:** 1 Department of Community Health Sciences, University of Manitoba, Winnipeg, Manitoba, Canada; 2 Department of Internal Medicine, University of Manitoba, Winnipeg, Manitoba, Canada; Universidade Federal de Pelotas, BRAZIL

## Abstract

**Background:**

The rising prevalence of multimorbidity poses challenges to health systems globally. The objectives of this study were to investigate: 1) the association between multimorbidity and depressive symptoms; and 2) whether social support plays a protective role in this association.

**Methods:**

A prospective population-based cohort study was conducted to analyze baseline and 3-year follow-up data of 16,729 community dwelling participants aged 65 and above in the Canadian Longitudinal Study of Aging (CLSA). Multimorbidity was defined as having three or more chronic conditions. The 10-item Center for Epidemiologic Studies Depression scale (CESD-10) was used to measure depressive symptoms. The 19-item Medical Outcomes Study (MOS) Social Support Survey was employed to assess perceived social support. Multivariate logistic regression models were used to examine the association between multimorbidity, social support and depressive symptoms.

**Results:**

Multimorbidity was very common among participants with a prevalence of 70.6%. Fifteen percent of participants had depressive symptoms at baseline. Multimorbidity was associated with increased odds of having depressive symptoms at 3-year follow-up (adjusted odds ratio, aOR = 1.51, 95% CI 1.33, 1.71), and developing depressive symptoms by follow-up among those with no depressive symptoms at baseline (aOR = 1.65, 95% CI 1.42, 1.92). Social support was consistently associated with decreased odds of depressive symptoms, regardless of level of multimorbidity.

**Conclusion:**

Multimorbidity was positively associated with depressive symptoms over time, but social support served as a protective factor. As a modifiable, protective factor, emphasis should be placed in clinical practice to assess social support and refer patients to appropriate services, such as support groups. Similarly, health policy should focus on ensuring that older adults have access to social support opportunities as a way to promote mental health among older adults. Community organizations that offer social activities or support groups play a key role in this respect and should be adequately supported (e.g., with funding).

## Introduction

Multimorbidity, the presence of multiple chronic conditions, is common, especially among older adults, affecting more than half of the older population [1]. The prevalence of multimorbidity is increasing due to longer life expectancy and the growing number of people who live with chronic conditions [2]. Multimorbidity is associated with negative consequences including higher disability, functional decline, cognitive impairment, decrease in quality of life, high health care costs and increased risk of death [3–9]. The relationship between multimorbidity and mental health has also been examined [10–12]. A meta-analysis found that people living with multimorbidity were three times more likely to have depression than those with no individual physical chronic condition, and two times more likely than those without multimorbidity [11]. However, all of the 40 studies included in the review were cross-sectional analyses, which did not allow assessment of whether morbidity preceded depression or vice versa. Relatively few longitudinal studies have examined the relationship between multimorbidity and depression among older adults [12]. We addressed this issue in the present study by examining whether multimorbidity predicts depressive symptoms prospectively in older Canadians. A second objective was to examine the role of social support in this relationship.

### Depression

Depression is one of the most common mental disorders, affecting about 7% of the world’s older population with a major impact on global public health [13]. Definitions and measures of depression vary widely in the literature and include assessments for major depression disorder using clinical diagnostic criteria; in epidemiologic studies, validated self-report instruments (e.g., Center for Epidemiologic Studies Depression Scale) are typically used to assess depressive symptoms. It has been estimated that between 8% to 16% of community dwelling adults aged 65 or older have clinically significant depressive symptoms, with prevalence estimates of major depression disorder ranging from1% to 4% [14].

Although depression is less prevalent in older adults than in younger individuals [15, 16], it is well known that late life depression has adverse consequences on health, such as an increased mortality risk due to suicide [14, 17], impaired executive function [18], increased disability [15], and poor quality of life [19]. Health care costs incurred for patients with both depression and chronic physical conditions are approximately 50% greater than for those with a chronic physical condition alone [20]. The clinical assessment of patients with both depressive symptoms and chronic medical conditions is complicated, and major depression frequently goes undetected and untreated among these individuals [21, 22]. Sometimes, depression among older adults may get overlooked due to atypical symptoms, or because it is considered a typical reaction due to aging [15, 23]. Moreover, treatment can be difficult. Antidepressants are found to be not as effective in older adults and are associated with increased complications and drug-disease interactions [24, 25]. These findings suggest the need to look for modifiable factors that can attenuate the risk of depression, including among older adults with multimorbidity. One such modifiable factor is social support [26–28].

### Social support and depression

Social support is one of the social determinants of health in the general population [29]. It represents the psychological and material resources individuals perceive to have available to them, or are actually receiving from their social network [30]. In older adulthood, the exchange of support is one of the most important functional components of an individual’s social network [31]. Social support is commonly categorized into three types: emotional, instrumental, and informational support [30, 32–35]. Emotional support refers to the things that people do that make the receiver feel being loved, trusted and cared for, which can bolster their sense of value and worth. Emotional support usually takes the form of non-tangible types of assistance and may involve expression of sympathy, caring, reassurance, and trust and provision of opportunities for emotional expression and venting. Instrumental support refers to various types of tangible help including financial assistance, or the provision of material goods, and services. Informational support refers to the help that others may offer through the provision of relevant information advice, guidance, and suggestions to help the individual solve problems.

The protective effects of social support in maintaining good health and decreasing vulnerability to physical and mental illnesses in older adults are well documented in the literature [15, 26–28, 35]. Lack of social support can be a risk factor for developing depressive symptoms in late life [15], and both adverse life events and poor perceived social support predict long-term major depressive disorder [36]. In a systematic review, over 90% of the 33 studies included found a significant association between social support and protection from depression, predominantly defined in terms of self-reported depressive symptoms, in older adults aged 50 years and older [26].

From a theoretical perspective, two explanations for why social support may be beneficial have been proposed in the psychological literature [34]. The direct effect model suggests that social support is overall beneficial by, for example, helping people regulate health behaviors and help with access to health care by providing informal resources (e.g., economic assistance, transportation) [34]. In contrast, the stress buffering model suggests that social relationships and the supports they provide are particularly beneficial in situations of stress, which consequently benefits health [34]. As such, social support may buffer against the deleterious effects of life stressors, such as illness, bereavement, crime, job loss, and social relationship crises [34, 37, 38]. The belief that others will provide support during challenging times may enhance one’s perceived ability and confidence to cope with demands, therefore leading to more positive appraisal of the situation and lower level of stress, with consequent benefits for health [34, 39]. The belief that needed social support is at hand (perceived social support) may also dampen the emotional and physiological responses to the event or alter maladaptive behavioral responses, such as by promoting health-related behaviors like exercise, practicing personal hygiene, healthy eating, and resting [34].

Research provides evidence for both the direct effect and stress buffering explanations of social support [34]. For example, in support of the stress buffer explanation, Ahn et al. in a study based on data from older adults aged 65 years or older from the 2006–2012 Health and Retirement Study in the US, found that positive spousal support attenuated the deleterious effect of multimorbidity on depression more, as the number of chronic conditions increased [40]. Similarly, focusing on older individuals with a single disease, another study showed that social support decreased depressive symptoms among those with severe arthritis, albeit not those with mild arthritis [41]. Yet other studies show an overall benefit of social support [34]. For example, among patients with rheumatoid arthritis, social support was associated with fewer depressive symptoms regardless of levels of pain [42] or functional ability [43]. The present study was designed to add to this literature on social support in the context of multimorbidity among older adults.

### The present study

This study had the following two objectives: (1) To examine the association between multimorbidity and depressive symptoms over time among Canadians aged 65 or older living in a community setting; and (2) to investigate the role of social support in this association. Regarding the association between multimorbidity and depressive symptoms, it was hypothesized that multimorbidity would be associated with a significantly increased likelihood of having depressive symptoms at follow-up. For the role of social support, we explored whether it would serve as a buffer against the negative effect of multimorbidity on depressive symptoms [34, 40, 41]. To the extent that multimorbidity functions as stressor, the stress buffer hypothesis would suggest that people with multimorbidity would benefit more from high levels of social support than those without multimorbidity; in other words, social support would be expected to reduce the likelihood of depressive symptoms among those with multimorbidity, but less so among those without multimorbidity.

## Methods

### Study design

This population-based prospective cohort study was a secondary analysis of data collected in the Canadian Longitudinal Study on Aging (CLSA).

### Data source

The baseline and first follow-up data of the CLSA were used for this study. CLSA is a large, Canada-wide population-based prospective cohort study. CLSA was designed to follow 51,338 community-dwelling Canadians, aged 45 to 85 at the time of recruitment for 20 years or until death [44–46]. CLSA is composed of two cohorts; both cohorts were used in the present study. The Comprehensive Cohort completes in-home interviews and visits a CLSA data collection site (DCS) for a wide range of assessments (e.g. physical, clinical); the Tracking Cohort is only surveyed via computer-assisted telephone interviews. The Comprehensive Cohort is composed of 30,097 participants who were randomly (within age/sex strata) recruited within 25–50 km of the 11 DCS (Victoria, Vancouver, Surrey, Calgary, Winnipeg, Hamilton, Ottawa, Montreal, Sherbrooke, Halifax, and St. John’s) in 7 Canadian provinces. The Tracking Cohort consists of a national, generalizable random sample of 21,241 participants who were selected within age/sex strata in each of the 10 Canadian provinces. The following groups were excluded from participation in the CLSA: people who could not communicate in one of the two official languages (English or French); those cognitively impaired at time of recruitment; full-time members of the Canadian Armed Forces; residents in a long-term care institution; and Aboriginal people living on reserves or other settlements at baseline [44–46].

As a national research platform, CLSA data covers a broad range of biological, medical, psychological, socioeconomic, and lifestyle factors that influence disease, health and well-being, which allows multidisciplinary research on physical, mental and social perspectives of healthy aging [44–46]. The CLSA’s major follow-ups are being carried out every three years. The baseline data were collected between 2011 and 2015; the first follow-up data collection started in 2015 and ended in 2018 [47]. Similar questionnaires to those in the baseline were used to collect data from both the Tracking and Comprehensive cohorts at follow-up.

### Study sample

**Fig 1:**
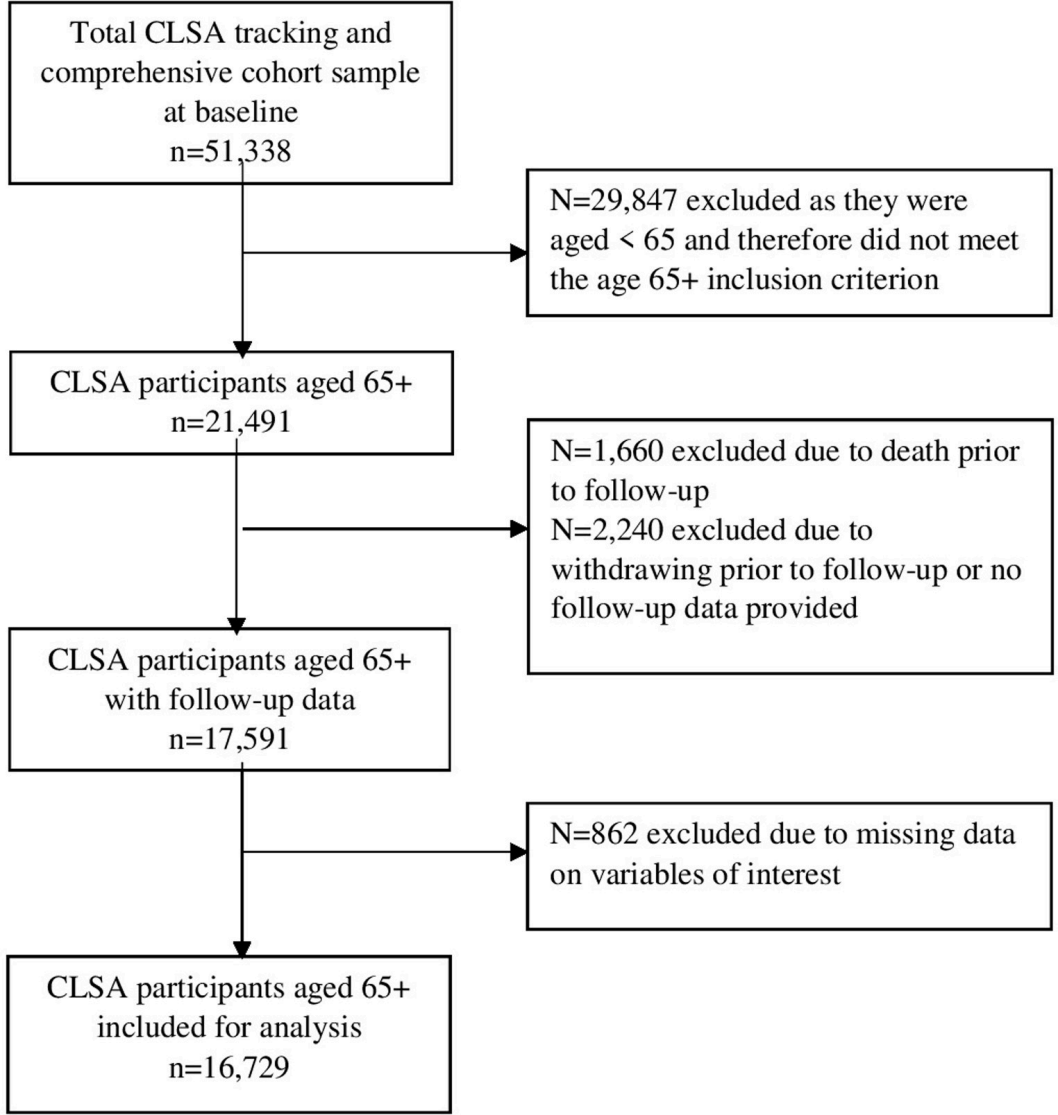
Study sample flowchart.

In the present study, we included participants aged 65+ at baseline in the Tracking and Comprehensive cohorts of the CLSA who provided questionnaire data at both baseline and follow-up 1. After excluding CLSA participants who were younger than 65 at baseline, 21,491 individuals remained (Fig 1). Of these, 17,591 had follow-up data. After excluding an additional 862 participants who had missing data on the variables used in this study, we were left with 16,729 participants for analysis. Chi-square analysis indicated that compared to participants included in the analysis, those aged 65+ who were excluded were more likely to be older, male, and married, were less likely to have postsecondary education, and had a lower household income. They were also more likely to have some functional impairment, 3 or more chronic conditions, and depressive symptoms at baseline.

### Study variables

#### Depressive symptoms

Depressive symptoms were measured in the CLSA at both the baseline and the three-year follow-up, using the 10-item Center for Epidemiologic Studies Depression scale (CESD-10) [48, 49]. This reliable and valid instrument is widely used to screen for depressive symptoms [50, 51]. In the questionnaire, participants are asked 10 questions regarding feelings of depression, loneliness, hopefulness for the future, restless sleep, etc., where each question has four response options: “all of the time”, “occasionally”, “some of the time”, “rarely or never”. Scores of the 10 questions were summed to generate a total score ranging between 0 and 30 with higher scores indicating greater symptom severity. In the present study, the CESD-10 scale was dichotomized using the validated cut-off score of 0–9 vs 10 or more [48]. A 10+ cut-off is widely used in research to identify people with depressive symptoms versus those who have fewer or no symptoms [52, 53].

#### Multimorbidity

CLSA participants were asked about their chronic conditions that lasted at least six months and had been diagnosed by a health professional. The CLSA questionnaire includes 42 questions of ten broad categories of chronic conditions: osteoarthritis (OA), arthritis, respiratory, cardiac/cardiovascular, neurological, gastrointestinal, vision, cancer, mental health, and other conditions. There is no one common definition of multimorbidity in the literature; rather, researchers have used different types of chronic conditions (including individual conditions and categories of conditions), varying numbers of conditions, and different multimorbidity cut-offs to classify people (e.g., 2+, 3+) [54–57].

After excluding acute issues, infectious diseases, cognitive and mental health diagnoses, and subjective symptoms (except low back pain), and consistent with previous research using CLSA data [58], we included 31 physical conditions in the present study: OA, rheumatoid arthritis (RA), other arthritis, Chronic Obstructive Pulmonary Disease (COPD), asthma, hypertension, diabetes, heart disease, angina, myocardial infarction (MI), peripheral vascular disease (PVD), cerebrovascular disease (CVD), transient ischemic attack (TIA), Parkinson’s disease, multiple sclerosis (MS), epilepsy, migraines, gastrointestinal ulcer, bowel disease (IBD) (which included Crohn’s disease, ulcerative colitis and irritable bowel syndrome), allergies, bowel incontinence, urinary incontinence, cataracts, glaucoma, macular degeneration, cancer, osteoporosis, back problems, hypothyroidism, hyperthyroidism, and kidney disease. An index variable for multimorbidity was created by summing across all chronic conditions and then dichotomizing the resulting score into two categories: ‘0’ = 0–2 chronic conditions present (reference) and ‘1’ = 3+ conditions. We chose this cut-off as the number of chronic conditions increases with age [58] and is more suitable for an older population than a 2 or more conditions cut-off [58–60].

#### Social support

The 19-item Medical Outcomes Study (MOS) Social Support Survey was used in the CLSA survey to assess perceived social support [61]. MOS contains four sub-scales of social support: affectionate support (3 items; involving expressions of love and affection. e.g. “someone who hugs you”); emotional/informational support (8 items; the expression of positive affect, empathetic understanding, and the encouragement of expressions of feelings, or the offering of advice, information, guidance or feedback. e.g. “someone you can count on to listen to you when you need to talk”); positive social interaction (4 items; the availability of other persons to positively interact with. e.g. “some to get together with for relaxation”); and tangible support (4 items; the provision of material aid or behavioral assistance. e.g. “someone to help you if you were confined to bed”). The MOS has good psychometric properties with high reliability: α = 0.91 for Affectionate Support, α = 0.92 for Tangible Support, α = 0.94 for Positive Social Interaction, and α = 0.96 for Emotional/Informational Support (Sherbourne & Stewart, 1991). In the questionnaire, participants were asked how often each type of support was available to them: “all of the time” (5), “occasionally” (4), “some of the time” (3), “rarely” (2), or “never” (1). A global score for perceived social support was created by first calculating the mean for each subscale and then taking the average of the scales. This resulted in a weighted score with a range from 1–5, with higher scores indicating a higher level of perceived social support available [62].

#### Covariates

Evidence shows that levels of depressive symptoms differ by age [63–65], sex [66], marital status [67], educational level [68], income level [69] and rural or urban area of residence [70]. These variables were therefore included as covariates in the analyses.

Age was grouped into two categories: aged 65–74, 75+ (reference). Sex included males (reference) and females. Marital status was grouped as married (married or living in common-law) and unmarried (single, never married or never lived with a partner, divorced or separated, or widowed; reference). Educational level was categorized as lower than postsecondary (elementary school, high school; reference) and at least some postsecondary (college graduate, Bachelor’s degree and postgraduate degree). Household income was assessed by asking, “What is your best estimate of the total household income received by all household members, from all sources, before taxes and deductions, in the past 12 months?” There are five categories in the CLSA data set: 1 = less than $20,000 (reference), 2 = $20,000 or more, but less than $50,000; 3 = $50,000 or more, but less than $100,000; 4 = $100,000 or more, but less than $150,000; and 5 = $150,000 or more. We retained these five categories as previous research has shown a dose-response relationship with depressive symptoms in the CLSA [71]. In addition, a “missing” category was created in order to keep those individuals (7.5%) who did not respond to this question in the analyses. Participants’ area of residence was provided in the CLSA dataset as: Rural; Urban core; Urban fringe; Urban population center outside a Census Metropolitan Area and Census Agglomeration; Secondary core; and Postal code link to dissemination area. This variable was recoded as 0 = rural (reference) and 1 = urban, with the latter including all non-rural categories.

Furthermore, we adjusted for functional status in the analyses. Functional status was assessed using the Older Americans’ Resources and Services (OARS) Multidimensional Functional Assessment Questionnaire including Activities of Daily Living (ADLs) and the Instrumental Activities of Daily Living (IADLs) [72]. The OARS scale has been validated [72], demonstrating high correlations with physical therapist measures of self-care capacity (r = 0.89). The reliability is high for both ADL (Spearman rho = 0.84) and IADL (Spearman rho = 0.87). The ADL focus on seven basic self-maintenance activities: dressing, eating, grooming, walking, getting out of bed, bathing, and toileting. The IADL focus on seven instrumental activities to support independent living within home and community: using a telephone, walking a distance, shopping, preparing meals, housework, taking medications, and managing money. Respondents indicate if they can do each activity: 1) without help; 2) with some help; or 3) unable to do the activity. From these variables, CLSA data includes a derived functional status measure based on ADL and IADL scores with 5 categories: no functional impairment, mild impairment, moderate impairment, severe impairment, and total impairment. As few CLSA participants had any functional impairment, we created a dichotomous variable: Those with no functional impairment were coded as “0” (reference); those with mild, moderate, severe, or total impairment were coded as “1”.

As recommended by CLSA when conducting weighted analyses, all ten Canadian provinces (Manitoba: reference) and type of cohort (1 = Comprehensive Cohort, 0 = Tracking Cohort, reference) were also included in the analysis as covariates [73].

### Statistical analysis

Weighted analyses were conducted to account for the complex sampling design of CLSA data and obtain unbiased estimates representing the Canadian population. The CLSA database contains two types of weights: trimmed weights and analytic weights. The trimmed weights reflect the number of participants in each sex, age group, education level and province, while the analytic weights are rescaled trimmed weights, which reflect the sample sizes within geographic strata [72]. Trimmed weights are recommended for descriptive statistics, analytic weights for inferential statistics [72].

The study cohort was described by means, standard deviations (SD), percentages and 95% confident intervals (CI) using the trimmed weights. A series of multivariate logistic regression models using the analytic weights were built to examine the association between multimorbidity and depressive symptoms (no depressive symptoms/at least some depressive symptoms) at follow-up. First, model 1 was created to estimate the association between multimorbidity and social support at the baseline and having depressive symptoms at follow-up, while controlling for baseline depressive symptoms. In model 2, we introduced a multimorbidity x social support interaction term to further test whether self-reported social support mitigates the risks of depressive symptoms associated with multimorbidity over time. We included age, sex, marital status, education, household income, functional status, and residence area, provinces and cohort, all derived from baseline data, as covariates in both models. Second, we selected participants who reported no or relatively few depressive symptoms (CESD-10<9) at baseline and repeated the two models to examine the relationship between multimorbidity and social support and developing depressive symptoms by follow-up.

We also conducted several sensitivity analyses. First, we reran the 2 models with 3 multimorbidity categories: 0–2, 3–4, and 5+ chronic conditions. Second, we created a change variable for chronic conditions (stable low/improved, worsened, stable high; see Results section for further details) and repeated the models. Third, we ran separate analyses by functional status (no functional impairment and mild/moderate/severe/total functional impairment, respectively).

All analyses were conducted using SAS version 9.4 (SAS Institute, Cary, NC). Statistical significance was assessed using a nominal α = 0.05.

### Ethics approval

CLSA received ethics approval from all institutions that participate in data collection. Participants provided written consent before participating in the study, and re-consented at each follow-up. The present secondary analysis of CLSA data received research ethics board approval from the University of Manitoba, and the approval to access the data from CLSA.

## Results

### Characteristics of the study population

The selected 16,729 study participants represented 3,178,274 Canadians aged 65 years and older. Table 1 presents the characteristics of the study population. For example, about two thirds of the study population were married (68.4%) and most had at least postsecondary education (76%). The majority lived in urban areas (78.6%) and had no functional impairment (86.6%). Less than one fifth (14.9%) had depressive symptoms (CESD-10 score of 10+) at baseline. More than two thirds (70.6%) of participants reported having 3+ chronic conditions at baseline. The average number of chronic conditions was 4.2 and most participants reported having a great amount of social support (mean 4.3). (Data not shown in table).

**Table 1:** Characteristics of study population at baseline*.

Variable	Total Population (Weighted N = 3,178,274)	Multimorbidity 0–2 Conditions (Weighted N = 933,126)	Multimorbidity 3+ Conditions (Weighted N = 2,245,148)	p value
	% (95% CI)	% (95% CI)	% (95% CI)	
**Age group**				p < .0001
65–74 years old	64.4 (63.2, 65.5)	76.2 (74.3, 78.1)	59.5 (58.1, 60.9)	
75+ years old	35.6 (34.3, 36.8)	23.8 (21.9, 25.7)	40.5 (39.1, 41.9)	
**Sex**				p < .0001
Female	54.0 (52.7, 55.3)	42.9 (40.6, 45.3)	58.6 (57.1, 60.0)	
Male	46.0 (44.7, 47.3)	57.1 (54.6, 59.4)	41.4 (39.9, 42.9)	
**Marital status**				p < .0001
Unmarried	31.6 (30.5, 32.7)	24.3 (22.4, 26.2)	34.6 (33.2, 35.9)	
Married	68.4 (67.2, 69.5)	75.6 (73.7, 77.6)	65.4 (64.1, 66.8)	
**Education**				p = .0024
Less than postsecondary	24.0 (23.0, 25.1)	22.5 (20.4, 24.5)	24.7 (23.4, 26.0)	
Postsecondary	76.0 (74.9, 77.0)	77.5 (75.5, 79.6)	75.3 (74.0, 76.6)	
**Household income**				p < .0001
< $20,000	5.8 (5.2, 6.3)	4.6 (3.6, 5.5)	6.2 (5.5, 6.9)	
$20,000 to < $50,000	34.6 (35.8, 37.5)	30.6 (28.4, 32.8)	36.3 (34.9, 37.7)	
$50,000 to < $100,000	37.4 (36.2, 38.7)	42.1 (39.7, 44.5)	35.5 (34.0, 36.9)	
$100,000 to < $150,000	9.7 (9.0, 10.5)	10.0 (8.6, 11.4)	9.6 (8.7, 10.5)	
$150,000 or more	4.9 (4.4, 5.5)	6.0 (5.6, 7.9)	4.5 (3.9, 5.1)	
No response	7.5 (6.9, 8.1)	6.7 (5.6, 7.9)	7.8 (7.1, 8.6)	
**Area of residence**				P<0.0001
Rural	21.4 (20.2, 22.5)	23.4 (21.1, 25.6)	20.5 (19.2, 21.9)	
Urban	78.6 (77.5, 79.8)	76.6 (74.4, 78.9)	79.5 (78.1, 80.8)	
**Functional status**				p < .0001
No functional impairment	86.6 (85.7, 87.4)	95.3 (94.4, 96.3)	82.9(81.8, 84.0)	
Mild, moderate, severe, total impairment	13.4 (12.6, 14.3)	4.7 (3.7, 5.6)	17.1(16.0, 18.1)	
**Depressive symptoms**				p < .0001
No (CESD-10<10)	85.1 (84.2, 86.0)	90.7 (89.2, 92.1)	82.8 (81.6, 83.9)	
Yes (CESD-10 = 10+)	14.9 (14.0, 15.8)	9.3 (7.9, 10.8)	17.2 (16.1, 18.4)	
**Multimorbidity**				-
0–2 conditions	29.6 (28.2, 30.5)			
3+ conditions	70.6 (69.5, 71.8)			

*Weighted numbers and percentages are derived using trimmed weights. P-values are derived from weighted chi-square analyses using analytic weights.

Participants with 3+ conditions differed on all variables from those with 0–2 conditions on baseline characteristics. For example, they were more likely to be older, female, unmarried, with a lower income, and have some functional impairment. They also reported having less social support (t = 7.56, p < .0001; data not shown in table).

### Association between multimorbidity and depressive symptoms

Table 2 presents results from the weighted analyses of a series of multivariate logistic regression models. Model 1 showed significant positive relationships between both multimorbidity and social support and depressive symptoms at 3-year follow-up. Participants with 3+ conditions had 1.5 times greater odds of having depressive symptoms at follow-up, compared to those with 0–2 conditions, whereas having more social support available was significantly related to reduced odds of depressive symptoms (aOR = 0.62). Covariates were also related to depressive symptoms at follow-up: younger age, postsecondary education, and a household income of $100,000 or more were associated with lower odds of depressive symptoms, whereas being female, married, functional impairment, and depressive symptoms at baseline increased the odds.

**Table 2:** Association between multimorbidity, social support and depressive symptoms 3 years later.

	Total Population	Multimorbidity 0–2 Conditions	Multimorbidity 3+ Conditions
Predictors	AOR (95% CI)	AOR (95% CI)	AOR (95% CI)
**Multimorbidity**			
0–2 conditions (ref)	-	-	-
3+ conditions	1.51 (1.33, 1.71)***	-	-
**Social support**	0.62 (0.58, 0.66)***	0.50 (0.43, 0.59)***	0.65 (0.60, 0.70)***
**Age group**			
75+ years old (ref)	-	-	-
65–74 years old	0.87 (0.79, 0.97)**	0.81 (0.63, 1.05)	0.89 (0.79, 0.99)*
**Sex**			
Male (ref)	-	-	-
Female	1.32 (1.19, 1.47)***	1.27 (0.99, 1.62)	1.34 (1.19, 1.51)***
**Marital status**			
Unmarried (ref)	-	-	-
Married	1.24 (1.10, 1.40)**	1.30 (0.97, 1.75)	1.23 (1,08, 1.40)
**Education**			
Less than postsecondary (ref)	-	-	-
Postsecondary	0.87 (0.77, 0.98)*	0.82 (0.62, 1.07)	0.88 (0.77, 1.01)
**Household income**			
<$20,000 (ref)	-	-	-
$20,000 to < $50,000	0.88 (0.73, 1.07)	0.99 (0.59, 1.68)	0.85 (0.69, 1.04)
$50,000 to < $100,000	0.69 (0.65, 1.02)	0.69 (0.40, 1.20)	0.68 (0.54, 0.85)**
$100,000 to < $150,000	0.62 (0.47, 0,81)**	0.59 (0.31, 1.13)	0.61 (0.46, 0.82)**
> $150,000	0.51 (0.37, 0.71)***	0.44 (0.20, 0.97)*	0.52 (0.36, 0.75)**
No response	0.83 (0.65, 1.05)	0.65 (0.34, 1.25)	0.85 (0.66, 1.10)
**Area of residence**			
Rural (ref)	-	-	-
Urban	1.07 (0.91, 1.25)	1.06 (0.75, 1.50)	1.07 (0.90, 1.28)
**Functional status**			
No Functional impairment (ref)	-	-	-
Mild, moderate, severe, total impairment	1.73 (1.52, 1.97)***	2.30 (1.50, 3.54)***	1.70 (1.49, 1.94)***
**Depressive symptoms at baseline**			
No (CESD-10 <10) (ref)	**-**	**-**	**-**
Yes (CESD-10 = 10+)	6.09 (5.44, 6.81)***	8.18 (6.19, 10.82)***	5.72 (5.07, 6.46)***

Note: Social support was treated as a continuous variable and ranged from 1–5. Analyses were also adjusted for type of cohort (comprehensive, tracking) and province of residence. P< *0.05; **0.01; ***0.001.

In a second model, we added the multimorbidity x social support interaction to the analysis. As the interaction was statistically significant (b = 0.29; SE 0.08, p = .0002), we further conducted separate analyses for those with 0–2 conditions and those with 3+ conditions, respectively (Table 2). Social support was significantly related to reduced odds of depressive symptoms among both 0–2 conditions and 3+ conditions groups (aOR = 0.50 vs 0.65).

Table 3 shows results for participants with a CESD-10 score of less than 10 at baseline. Among these individuals, 10.1% developed depressive symptoms by follow-up (i.e., had a CESD-10 score of 10+ at follow-up). Again, having 3+ conditions increased the odds of developing depressive symptoms (aOR = 1.65), whereas social support decreased the odds (aOR = 0.61). Moreover, a significant interaction was again observed (b = 0.25; SE = 0.09, p = .0062). Separate analyses for those with 0–2 conditions and those with 3+ conditions (6.2% of participants with 0–2 conditions had developed at least some depressive symptoms by follow-up, compared to 11.7% among those with 3+ conditions) also showed statistically significant effects for social support (Table 3).

**Table 3:** Association between multimorbidity, social support and developing depressive symptoms (CESD = 10+) by 3-year follow-up.

	Total Population	Multimorbidity 0–2 Conditions	Multimorbidity 3+ Conditions
Predictors	AOR (95% CI)	AOR (95% CI)	AOR (95% CI)
**Multimorbidity**			
0–2 conditions (ref)	-	-	-
3+ conditions	1.65 (1.42, 1.92)***	-	-
**Social support**	0.61 (0.56, 0.66)***	0.52 (0.43, 0.62)***	0.63 (0.57, 0.69)***
**Age group**			
75+ years old (ref)	-	-	-
65–74 years old	0.78 (0.69, 0.88)**	0.75 (0.56, 0.99)*	0.78 (0.68, 0.89)**
**Sex**			
Male (ref)	-	-	-
Female	1.43 (1.26, 1.63)***	1.55 (1.17, 2.06)**	1.41 (1.22, 1.63)***
**Marital status**			
Unmarried (ref)	-	-	-
Married	1.26 (1.08, 1.46)**	1.10 (0.78, 1.54)	1.30 (1.10, 1.53)**
**Education**			
Less than postsecondary (ref)	-	-	-
Postsecondary	0.90 (0.78, 1.04)	0.90 (0.66, 1.23)	0.89 (0.76, 1.05)
**Household income**			
<$20,000 (ref)	-	-	-
$20,000 to < $50,000	0.87 (0.69, 1.11)	1.13 (0.62, 2.07)	0.81 (0.63, 1.06)
$50,000 to < $100,000	0.68 (0.52, 0.88)**	0.80 (0.42, 1.53)	0.64 (0.49, 0.85)**
$100,000 to < $150,000	0.62 (0.45, 0,85)**	0.61 (0.28, 1.34)	0.61 (0.43, 0.87)**
> $150,000	0.48 (0.32, 0.72)**	0.42 (0.16, 1.11)	0.50 (0.32, 0.78)**
No response	0.79 (0.59, 1.06)	0.68 (0.31, 1.48)	0.81 (0.59, 1.12)
**Area of residence**			
Rural	-	-	-
Urban	1.07 (0.91, 1.25)	1.06 (0.75, 1.50)	1.07 (0.90, 1.28)
**Functional status**			
No functional impairment (ref)	-	-	-
Mild, moderate, severe, total impairment	1.73 (1.47, 2.02)***	2.50 (1.55, 4.04)**	1.68 (1.42, 1.98)***

Note: Social support was treated as a continuous variable and ranged from 1–5. Analyses included only those participants with CESD-10 scores <10 at baseline. Analyses were also adjusted for type of cohort (comprehensive, tracking) and province of residence. P< *0.05; **0.01; ***0.001.

### Sensitivity analyses

We conducted a first sensitivity analysis by dividing participants into three chronic condition groups using tertiles (0–2 condition, 3–4 conditions, and 5+ conditions) and repeating the logistic regression analyses (Table 4A). Participants with 3–4 conditions had 1.28 the odds of depressive symptoms 3 years later, and those with 5+ conditions had 1.72 greater odds, relative to those with 0–2 conditions. Social support again reduced the odds of depressive symptoms. Adding interaction effects between levels of multimorbidity and social support revealed a significant effect for the 3–4 conditions x social support interaction (b = 0.27, p < .01), but not for the 5+ conditions x social support interaction (b = .30, p>.05). Stratifying analyses by the 3 multimorbidity showed that for each group, social support significantly reduced the odds of depressive symptoms: aOR = 0.50 (CI 0.43, 0.59), p < .0001 for 0–2 conditions; aOR = 0.63 (CI 0.56, 0.72), p < .0001 for 3–4 conditions; and aOR = .65 (CI 0.59, 0.72), p < .0001 for 5+ conditions.

**Table 4:** Association between multimorbidity, social support and depressive symptoms: 3 multimorbidity groups.

Predictors	AOR (95% CI)
**A. Multimorbidity– 3 groups**	
0–2 conditions (ref)	-
3–4 conditions	1.28 (1.11, 1.47)**
5+ conditions	1.72 (1.50, 1.97)***
**Social support**	0.62 (0.57, 0.66)***
**B. Multimorbidity–change over time**	
Stable low/improved (ref)	-
Stable high	1.56 (1.34, 1.82)***
Worsened	1.22 (0.99, 1.49)
**Social Support**	0.62 (0.58, 0.66)***

Note: Social support was treated as a continuous variable and ranged from 1–5. Analyses were adjusted for age group, sex, marital status, education, household income, area of residence, functional status, depressive symptoms at baseline, type of cohort (comprehensive, tracking), and province of residence. P< *0.05; **0.01; ***0.001.

As a second sensitivity analysis, we derived a new variable based on the change in chronic conditions from baseline to follow-up, three years later. In this sample, 14.6% of participants were ‘stable low’ as they had 0–2 chronic conditions at both baseline and follow-up; 3.9% ‘improved’ in that they had 3+ conditions at baseline, but only 0–2 at follow-up; 12.6% ‘worsened’ as they had 0–2 conditions at baseline, but 3+ at follow-up; and 68.9% were ‘stable high’, with 3+ conditions at both baseline and follow-up. As few participants had improved, we combined them with the ‘stable low’ group and created 3 categories: 1) stable low/improved (reference), 2) stable high, and 3) worsened, and repeated the regression analyses (Table 4B). Compared to the stable low/improved group, the stable high group had significantly higher odds of depressive symptoms (aOR = 1.56). The effect for the worsened group was not significant. Social support remained a significant protective factor (aOR = 0.62). Adding interaction effects showed a significant worsened x social support effect (b = -.39, p < .01, but not stable high x social support interaction (b = .02, p>.05). However, when conducting separate analyses for each group, respectively (stable low/improved, stable high, worsened), social support remained a significant protective factor for depressive symptoms for all groups: aOR = 0.66 (CI 0.54, 0.81), p < .0001 for the stable low/improved group; aOR = 0.64 (CI 0.59, 0.70), p < .0001 for the stable high group, and aOR = 0.43 (CI 0.34, 0.53), p < .0001 for the worsened group.

Additional sensitivity analyses were further conducted by functional status (for those without functional impairment and those with functional impairment, respectively) (Table 5). Multimorbidity remained a significant predictor of depressive symptoms among those with no functional impairment, but not those with functional impairment. Social support was a protective factor in both groups, reducing the odds of depressive symptoms at follow-up.

**Table 5:** Sensitivity analyses: Association between multimorbidity, social support and depressive symptoms among participants with and without functional impairment.

	No Functional Impairment	Mild, moderate, severe, total impairment
Predictors	AOR (95% CI)	AOR (95% CI)
**Multimorbidity**		
3+ conditions vs 0–2 conditions	1.54 (1.35, 1.76)***	1.15 (0.78, 1.70)
**Social support**	0.62 (0.57, 0.67)***	0.61 (0.53, 0.71)***

Note: Social support was treated as a continuous variable and ranged from 1–5. Analyses were adjusted for age group, sex, marital status, education, household income, area of residence, functional status, depressive symptoms at baseline, type of cohort (comprehensive, tracking), and province of residence. P< *0.05; **0.01; ***0.001.

## Discussion

The results of this population-based prospective cohort study contributes to the growing body of literature on multimorbidity by providing evidence that while multimorbidity increased the odds of depressive symptoms, social support served as a protective factor among older adults in a community setting. Moreover, the protective effect of social support was consistent across population groups, including among participants with different numbers of chronic conditions (0–2, 3 or more, and 5 or more), and those with no functional impairment or functional impairment.

The current research confirms the findings of previous cross-sectional studies on the association between multimorbidity and depressive symptoms [11]. Our results are also consistent with the relatively few existing longitudinal studies that show that multimorbidity is associated with incident depressive symptoms as well as persistence of depressive symptoms over time among older adults in a community setting in a range of countries, including the Netherlands, Taiwan, and China [12]. The mechanisms underlying the association between multimorbidity and depressive symptoms are not well-understood, but likely include a variety of biological, psychosocial, and care-related factors [12]. For example, research suggests that multimorbidity is disproportionally high among individuals with disadvantaged socio-economic status, disability, pain, or cognitive impairment conditions, which may increase the risk of mental health conditions like depression [74–79]. The relationship is also likely reciprocal in nature, with depression predicting multimorbidity, and multimorbidity, in turn, predicting the development of multimorbidity [12].

The present findings are also consistent with previous research that has documented the protective effect of social support in relation to depression [26–28]. However, our study does not support the stress buffer hypothesis [34], for which we might have expected social support to benefit those with more chronic conditions, but not those with few or no conditions. Indeed, social support attenuated depressive symptoms at all levels of multimorbidity, and even slightly more so among those with 0–2 chronic conditions. Social support also had a protective effect regardless of whether the number of chronic conditions worsened or remained stable over time. Thus, overall, our findings are more consistent with the direct effect social support hypothesis [34]. Our findings are, therefore, consistent with some previous research [34, 42, 43], albeit not all [40]. An important difference between the present study and previous research that found a buffering effect in the context of multimorbidity [40] is that we used an overall measure of social support. Previous research showed a buffer effect for positive support from a spouse only, but not support from other individuals [40]. This suggests that the specific type of social support, or the person providing the support, may make a difference. Future longitudinal studies should further examine this issue. It must also be recognized that, in terms of multimorbidity, we classified individuals as having 0–2 chronic conditions versus 3 or more conditions. This means that both groups contained people with multiple chronic conditions. Future research would be useful that would tease out the role of social support among those with no or only one chronic condition.

Social support was also protective in reducing the likelihood of developing depressive symptoms in the present study, being associated with reduced odds among those who were classified as having no or few depressive symptoms at baseline. Furthermore, having more social support was significantly associated with lower odds of depressive symptoms at follow-up among individuals with functional impairment, as well as among those without functional impairment. In other words, the findings were very consistent across different sub-population groups in our study.

Several other findings in our study warrant mention at this point. In general, being younger and having a household income of $50,000 or more decreased the odds of depressive symptoms at 3-year follow-up, as well as the odds of developing depressive symptoms among those with no or few depressive symptoms at baseline. Conversely, being female and having some functional impairment increased the odds. These effects generally emerged only in analyses for the total population, and among those with three or more chronic conditions, but not among those with zero to two conditions. This suggests that healthcare providers dealing with older adults with multiple chronic conditions should particularly monitor the mental health of female patients, those who live on live on low income, and those with functional limitations.

Noteworthy is also our finding that being married was associated with increased odds of depressive symptoms. This is contrary to some previous research that shows that being married is protective against depression [80. 81]. However, the relationship between marital status and mental health is complex and can vary by whether people have always been single, are divorced or widowed; findings also differ depending on sex and age [81]. Whether marriage affords health benefits further depends on marital quality or spousal support [82]. In the present study, we classified CLSA participants as either married or not married, but did not differentiate between those who were single, divorced or separated, or widowed. Examining how each of these categories relate to depressive symptoms, as well as how they interact with other variables such as sex, income, or social support is a task for future research.

Our findings have several implications. First, social support is an important factor to consider in reducing the risk of depression among older adults. Social support is derived from the social network that people are embedded in [38]. The complexities of who among social network members (e.g., close family, friends) provides which type of support (e.g., instrumental, emotional) have been described in the literature [38]. For example, while family members, particularly spouses, would be expected to provide the most social support and more peripheral social network members (e.g., co-participants at activity programs) might provide companionship, but not instrumental support [38, 83], this is not always the case. Not all marriages or partnerships provide a supportive environment [84], and friends may provide a wide range of social support, including instrumental support. Research indicates that having a diverse social network consisting of family, friends, but also neighbors and acquaintances provides the most opportunity to have various social support needs met; a restricted social network, in contrast, is associated with deficiencies in all types of support [85]. This means that it is important for older adults to retain a diverse social network, for example, by being able to participate in social activities at a community center, or staying in touch with family and friends. Community organizations play a key role in facilitating these opportunities by providing programs or other services, such as transportation options for those with mobility problems. For example, many communities and cities in Canada have active living centers for older adults that provide a variety of opportunities for socializing, physical activity, and lifelong learning.

A second implication is that, given the high prevalence of multimorbidity, clinical treatment for older adults with multimorbidity should take into consideration all conditions, rather than treating each condition in isolation. Importantly, mental health should be part of assessments and treatment approaches. As has been pointed in the literature, the healthcare system needs to be re-structured to count past one [86]. Clinicians providing care for older adults with multimorbidity should also assess the presence of perceived social support. Appropriate interventions or recommendations aimed at increasing social support should be considered to improve the mental health of older adults. For example, referrals to support groups offered by healthcare and community organizations, and community organizations that promote social bonds and create networking opportunities should be considered. Also, behavioral therapists can help to increase older individuals’ social support through teaching of skills directed at gaining support when needed [87, 88].

A third implication relates to policy. Given that the present study shows that both multimorbidity and social support play a role in depressive symptoms efforts should, on the one hand, be made to reduce the occurrence of multimorbidity through primary prevention, such as by mitigating the effects of low income, which is highly related to multimorbidity [10], via policy solutions that reduce socioeconomic inequalities [89]. On the other hand, the importance of social support needs to be recognized by policy makers. Ideally, this would mean adequately supporting (e.g., providing funding) community organizations that provide older adults with opportunities for social support, such as organizations that provide social activities or support groups. Given that older adults living on low income are at higher risk of experiencing both multimorbidity [10] and, as shown in the present study, depressive symptoms, programs need to be kept affordable to ensure that those most at risk can benefit from them.

### Strengths and limitations

This study has several strengths. A primary strength relates to the prospective nature and nationally generalizable sample used. This longitudinal study used the first follow-up data of CLSA of Canadians aged 65 or above, which allowed us to examine the relationship between multimorbidity, social support and depressive symptoms three years later, in a large nationally generalizable sample while controlling for demographic variables, which minimizes the population bias. Additionally, CLSA includes validated instruments such as the CESD-10 to measure depressive symptoms and the MOS to measure social support. In addition, relatively larger numbers (31) of chronic conditions were captured while measuring multimorbidity. In contrast, many of the previous studies were based on only a limited number of chronic conditions.

However, when interpreting the results of this study, several limitations should be considered. First, chronic conditions and depressive symptoms were self-reported by participants, raising the possibility of misclassification due to recall bias. Moreover, the social support measure assesses perceptions of the availability of social support, which may differ from the actual support available. Also, while the MOS scale is a commonly used tool, it does not provide information on who provides support, information that may be important in the context of depressive symptoms [40]. Second, the CLSA data do not include people living on First Nations reserves, full-time members of the Canadian Forces, and those living in long-term care facilities. In addition, the CLSA also does not include individuals who do not speak French or English and those with cognitive impairment, who may have lower social support, and may be more at risk for depression. Third, multimorbidity was assessed by simply counting the total number of conditions; CLSA does not include measures of disease severity. As such, all chronic conditions were treated equally and using a simple presence/absence dichotomy. There is also no information on whether a condition was successfully treated and cured. We also did not examine the relationship between specific chronic condition clusters and depressive symptoms.

Fourth, CESD-10 was used which, although it is commonly used to screen for depression, is not a diagnostic tool to determine clinical depression [90]. Another concern is the issue of symptom contamination in the CES-D, although CES-D has acceptable screening accuracy in the general population or primary care settings. Clearly somatic symptoms of depressive symptoms overlap with disease-related symptoms, particularly decreased energy, concentration difficulties, sleep, and appetite disturbance. Additionally, among participants who exhibited depressive symptoms at follow-up, we do not know when the onset of symptoms occurred during the follow-up period.

We also did not explore other possible changes from baseline to follow-up, such as change in marital status, which could affect social support and, consequently, depressive symptoms. Similarly, functional status could have changed over time. The impact of such possible changes should be explored in future research. Furthermore, while there was a relatively low rate of deaths at follow-up, and the competing risk of death was likely low in the cohort overall, it could be high among older participants in the group of non-respondents in this study. Our findings may also be affected by participant attrition due to loss at follow-up, and missing values on the measures included in this study. Thus, our findings may only be generalizable to relatively healthy older adults.

## Conclusions

In this population-based longitudinal cohort study we demonstrated a positive relationship between multimorbidity and depressive symptoms over a 3-year period among older adults aged 65 or older living in a community setting, with social support acting as a protective factor regardless of the number of chronic conditions. Future research would usefully explore additional issues, such as the possible mediating role that functional impairment or pain may play in the relationship between multimorbidity, social support, and depressive symptoms. There is also a need for intervention research focusing on how to best increase social support to improve the mental health of older adults living in the community, including among those with multimorbidity.
